# Chiglitazar Activates PPAR-α/γ to Suppress Oxidative Stress and Angiogenesis in Corneal Neovascularization

**DOI:** 10.3390/antiox15040449

**Published:** 2026-04-02

**Authors:** Tao Tao, Jiyuan Ye, Ruifeng Li, Yan Ke, Xiaoqin Zheng, Qinghe Zhang, Lan Zheng, Shuwen Wang, Zhen Zhang, Le Wang, Cheng Li

**Affiliations:** 1 Eye Institute & Affiliated Xiamen Eye Center, School of Medicine, Xiamen University, Xiamen 361102, China; 2 Department of Digestive Disease, Institute for Microbial Ecology, School of Medicine, Xiamen University, Xiamen 361004, China; 3 Department of Ophthalmology, The First Affiliated Hospital, University of South China, Hengyang 421001, China; 4 Huaxia Eye Hospital of Quanzhou, Quanzhou 362000, China; 5 Shen Zhen Research Institute, Xiamen University, Shenzhen 518057, China; 6 Fujian Provincial Key Laboratory of Ophthalmology and Visual Science, Xiamen 361102, China

**Keywords:** chiglitazar, corneal neovascularization, PPAR, multi-omics

## Abstract

Purpose: Chiglitazar (Chi) is a pan-peroxisome proliferator-activated receptor (PPAR) agonist with reported anti-oxidative effects in metabolic disorders. In this study, we investigate its therapeutic effects and potential mechanisms in corneal neovascularization (CNV). Methods: Scratch-wound and tube formation assays in human umbilical vein endothelial cells (HUVECs) were performed to evaluate the effects of Chi under recombinant human vascular endothelial growth factor (VEGF) stimulation. An oxidative stress model was established in human corneal epithelial cells (HCEs), and intracellular reactive oxygen species (ROS) levels were quantified by flow cytometry. A corneal alkali burn mouse model of CNV was established. Chi was then administered and compared with vehicle, pioglitazone, or fenofibrate. Corneal epithelial healing and neovascularization were assessed. Public drug–disease–target resources were integrated with RNA-seq data and single-cell transcriptomes to prioritize Chi-associated targets and pathways, which were examined by immunofluorescence, RT-PCR, and Western blotting. Ocular safety was evaluated by comprehensive ophthalmic evaluation. Results: Chi significantly inhibited migration and tube formation in VEGF-induced HUVECs, and flow cytometry confirmed effective ROS reduction. In vivo, Chi markedly improved corneal conditions compared with the vehicle and showed efficacy comparable to or superior to selective PPAR-α/γ agonists, depending on the outcome measures. Bioinformatic analyses predicted PPAR-γ as the dominant isoform, with PPAR-α secondary and PPAR-δ appearing less prominent, collectively implicating oxidative stress and VEGF pathways. Immunofluorescence verified PPAR-γ activation, predominantly localized to the corneal epithelium. RT-PCR and Western blotting supported activation of antioxidant pathways and suppression of angiogenic signals, with Western blotting confirming PPAR-γ and PPAR-α activation, whereas PPAR-δ activation appeared less evident under the present conditions. Ocular examinations demonstrated a favorable safety profile. Conclusions: Chi primarily activates PPAR-γ and PPAR-α, producing antioxidant and anti-angiogenic benefits, supporting its potential as a multi-target PPAR therapy for CNV.

## 1. Introduction

Corneal neovascularization (CNV) is a pathological change shared by multiple ocular surface diseases [1], including infectious keratitis [2], chemical burns [3], and pterygium [4]. Its development disrupts the cornea’s immune privilege and transparency [5], and in severe cases may lead to irreversible visual impairment or even blindness [6]. Current evidence indicates that CNV progression is regulated by multiple factors, among which oxidative injury and aberrant activation of the vascular endothelial growth factor (VEGF) signaling pathway are recognized as key contributors [7].

Oxidative damage plays an essential regulatory role in the expression and activity of angiogenic mediators [8]. Reactive oxygen species (ROS) upregulate the transcription of VEGF and enhance the affinity of its receptors, thereby promoting neovessel formation [9]. At the same time, oxidative stress can initiate or exacerbate inflammatory responses, sustaining the pathological angiogenic milieu [10]. VEGF is widely acknowledged as a central pro-angiogenic factor in CNV [11]. Among its family members, VEGF-A is the most prominent isoform and induces limbal vascular endothelial cell proliferation, migration [12], and invasion into the cornea primarily through activation of VEGFR-2 [13]. Thus, in addition to addressing the underlying ocular disease, therapeutic strategies for CNV should aim to simultaneously suppress oxidative stress and interrupt VEGF signaling to achieve optimal clinical benefit.

In recent years, therapeutic development for CNV has advanced considerably, particularly strategies targeting peroxisome proliferator-activated receptors (PPAR), which have gained attention for their dual capacity to modulate oxidative stress and angiogenesis [14]. PPAR-γ agonists, such as pioglitazone and rosiglitazone, have demonstrated anti-oxidative, anti-inflammatory, and anti-angiogenic activities across multiple studies [14], positioning them as promising candidates for CNV management. The PPAR-α agonist fenofibrate exhibits anti-inflammatory, anti-apoptotic, anti-oxidative, and anti-angiogenic effects [15], making it one of the PPAR-targeted agents with the most substantial support in this field. Research on PPAR-δ agonists remains more limited; available in vivo and in vitro data suggest potential in mitigating oxidative stress and inflammation [16]. Moreover, targeting a single PPAR isoform remains inadequate for managing complex or severe CNV.

Building on this background, Chiglitazar (Chi) is a novel PPAR modulator with dominant activation of PPAR-γ and additional activity toward PPAR-α and PPAR-δ. It is clinically approved for the treatment of type 2 diabetes [17]. Its multi-isoform activation profile provides a theoretical advantage in simultaneously regulating oxidative stress and angiogenic pathways, suggesting that Chi may possess unique therapeutic potential for CNV. Based on these considerations, we propose Chi as a promising candidate for CNV therapy, with the expectation that its dual modulation of oxidative injury and pathological vessel growth may address key limitations of existing treatments.

This study aims to evaluate the therapeutic efficacy of Chi in CNV using both in vitro experiments and an alkali burn-induced mouse model. In addition, we employed an integrated experimental–computational approach to preliminarily explore its mechanism of action, focusing on its multi-PPAR activation properties. Our findings may provide a basis for the future clinical translation of Chi as a potential therapeutic strategy.

## 2. Methods

### 2.1. Bulk RNA-Seq Analysis and Single-Cell Analysis

RNA-seq datasets (SRR13449163, SRR13449162, SRR13449161, SRR13449160, SRR13449159, and SRR13449158) were retrieved from the SRA database [18] using the Wget command-line tool. The raw sequencing files were processed following a standard bioinformatic pipeline [18]. Briefly, an initial quality assessment was performed with FastQC (v0.11.9), and adaptor contamination or low-quality reads were trimmed using Trimmomatic (v0.39). The cleaned reads were aligned to the mouse reference genome using HISAT2 (v2.2.1), followed by transcript assembly and quantification with StringTie (v2.2.1). Differentially expressed genes were identified with DESeq2 (v1.36.0) [19]. Gene set enrichment analysis (GSEA) was subsequently conducted in R (v4.5.0) using the Cluster Profiler package (v4.4.4) [20].

Single-cell transcriptome data were obtained from the publicly accessible dataset generated by the laboratory of Joshua R. Sanes (GSE199013) [21] through the Single Cell Portal (https://singlecell.broadinstitute.org/single_cell, accessed on 1 January 2024). The dataset was preprocessed according to the platform’s recommended workflow and used for downstream visualization and comparative analysis.

### 2.2. Cell Culture and Reagents

Human corneal epithelial cells (HCEs) were kindly provided by the RIKEN BioResource Center (Tsukuba, Japan). Cells were maintained in Dulbecco’s Modified Eagle Medium/Nutrient Mixture F-12 (DMEM/F-12), from Gibco (Grand Island, NY, USA) supplemented with 10% fetal bovine serum (FBS, Gibco) and 100 U/mL penicillin–streptomycin (Gibco). Human umbilical vein endothelial cells (HUVECs) were obtained from the American Type Culture Collection (ATCC, Manassas, VA, USA). HUVECs were cultured in medium containing 5% FBS, 1% penicillin–streptomycin (PS), and 1% endothelial cell growth supplement (ECGS). All cells were maintained in an incubator at 37 °C with 5% CO_2_ to ensure cellular viability and experimental consistency.

Anti-VEGF-A antibody (ab46154, Abcam, Cambridge, UK), anti-Lectin (I21413, Thermo, Waltham, MA, USA), PPAR-γ polyclonal antibody (I21413, Thermo, Waltham, MA, USA), Nrf2 polyclonal antibody (33123-1-AP, Wuhan, Proteintech), PPAR-δ rabbit antibody (A5656, Abclonal, Wuhan, China), recombinant human VEGF protein (HZ-1038, Proteintech), and PPAR-α rabbit antibody (A25296, Abclonal) were used in this study. Chi, fenofibrate, and pioglitazone (Nebula Bioscience, Beijing, China) were dissolved in dimethyl sulfoxide (DMSO). The solution was stored at −80 °C and freshly diluted to the desired working concentration immediately before use.

### 2.3. Cell Viability and Scratch Wound Healing Assays

Cells were seeded in 96-well plates at a density of 1 × 10^4^ cells per well. When the cell confluence reached approximately 80%, the culture medium was replaced with medium containing different concentrations of Chi and incubated for 24 or 48 h. Then, 10 μL of CCK-8 reagent (Beyotime, Shanghai, China) was added to each well and incubated for 1–2 h in the dark. Absorbance at 450 nm was measured using a microplate reader (Thermo, USA).

HUVECs were seeded in 6-well plates, and when the cells reached nearly 100% confluence, a vertical scratch was made using a 200 μL pipette tip. The wells were washed with PBS three times to remove cell debris, and serum-free medium containing different concentrations of Chi was added. Images at 0, 12, and 24 h were captured at the same field of view. Scratch areas were analyzed using ImageJ (v1.45g), and the migration rate was calculated based on changes in wound area.

### 2.4. Tube Formation Assay

One day prior to the experiment, 48-well plates and 200 μL pipette tips were pre-cooled at −20 °C. Matrigel was removed from −20 °C, placed on ice, and allowed to thaw slowly overnight at 4 °C. A total of 100 μL of pre-cooled Matrigel was added to each well of the chilled 48-well plate. After the gel naturally spread across the bottom, the plate was transferred to a 37 °C incubator for polymerization.

Cells from each treatment group were harvested with trypsin, resuspended in serum-free ECM medium, and adjusted to a density of 5 × 10^4^ cells/mL. Then, 100 μL of the cell suspension was carefully added to each Matrigel-coated well and incubated at 37 °C in 5% CO_2_ for 6 h. Tube formation was observed and imaged under a light microscope.

### 2.5. Flow Cytometric Analysis

According to the instructions of the ROS assay kit (Beyotime, Shanghai, China), the probe-loading method was used after collecting the cells. Specifically, HCEs were co-incubated with H_2_O_2_ (250 μM) and different concentrations of Chi for 6 h. After treatment, the cells were collected and mixed with the DCFH-DA probe and then incubated in a 37 °C cell incubator for 20 min, with gentle inversion every 3–5 min. The cells were then washed three times with sterile PBS to avoid interference. Finally, the cell suspension was filtered through a 70-μm cell strainer and analyzed using a CytoFlex S flow cytometer (Beckman Coulter, Brea, CA, USA).

### 2.6. Corneal Alkali Burn Model

Male C57BL/6 mice (6–8 weeks old, 25–35 g) were obtained from the Xiamen University Laboratory Animal Center (Xiamen, China). All procedures complied with the ARVO Statement for the Use of Animals in Ophthalmic and Vision Research. General anesthesia was achieved by intraperitoneal injection of 1% sodium pentobarbital (0.1 mL/100 g). Compound tropicamide was applied to induce mydriasis, followed by 0.5% proparacaine hydrochloride to ensure corneal surface anesthesia. A 2 mm circular filter paper was prepared in advance and saturated with 1 M NaOH (2 μL). The soaked disk was gently positioned on the central corneal surface for 20 s. Immediately afterwards, the ocular surface was rinsed with sterile saline for 20 s to remove residual alkali. Levofloxacin eye drops were administered at the end of the procedure to reduce the risk of infection and to maintain surface hydration. Corneal neovascular outgrowth was examined on days 1, 2, 5, and 8 after injury using slit-lamp microscopy, and images were archived for analysis.

The vascular length index was calculated using the formula I = (L/R) × 100%. The thickest and longest vessel growing toward the central cornea was selected as the representative vessel, with the distance from the limbus to the vessel tip recorded as L. In the same image, the maximal vertical corneal height was measured and recorded as R. The cornea was divided into four quadrants, and the neovessel lengths in each quadrant were measured and noted as l_1_, l_2_, l_3_, and l_4_. The vascularized area (S) was calculated using the following formula: S = 3.1416 × [(r^2^ − (r − l_1_)^2^) + (r^2^ − (r − l_2_)^2^) + (r^2^ − (r − l_3_)^2^) + (r^2^ − (r − l_4_)^2^)]/4, where r represents the corneal radius. The neovascularization index (i) was then determined as: i = (S/(3.1416 × r^2^)) × 100%.

### 2.7. Topical Treatment and Fluorescein Sodium Staining

Two independent in vivo experiments were performed. In the first experiment, animals were randomly assigned to three groups: control (ctrl), vehicle, and 100 μM Chi. In the second experiment, animals were assigned to four treatment groups: vehicle, 100 μM Chi, 200 μM fenofibrate, and 200 μM pioglitazone. The control group received no treatment. The vehicle group was given a mixture of PBS and 0.1% DMSO, a vehicle formulation commonly used in topical ocular studies and generally considered safe [22,23]. Chi, fenofibrate, and pioglitazone were diluted in PBS to prepare eye drop solutions, which were protected from light throughout use. All treated groups received topical administration of 5 μL per eye, three times daily.

To evaluate corneal wound healing, mice were anesthetized at the designated time points, and 2.5 μL of 10 mg/mL fluorescein sodium was applied to the ocular surface. After 10 s, the dye was removed by rinsing with PBS, followed by gentle blotting of any residual dye around the eye with clean filter paper. Corneal images were then obtained under a slit-lamp microscope, using both white-light and blue-light settings, to assess epithelial recovery.

Corneal opacity was scored from 0 to 4 as follows: 0, transparent cornea; 1, very slight opacity; 2, slight opacity with visible pupil and iris vessels; 3, moderate opacity with visibility of the pupil only; and 4, severe opacity with no visible anterior segment [7].

### 2.8. In Vivo Corneal Confocal Imaging

Following anesthesia, a thin layer of carbomer gel was applied evenly across the corneal surface to maintain hydration and minimize mechanical irritation. Corneal imaging was then performed using an in vivo corneal confocal microscope, focusing on the distribution, extension, and caliber of newly formed vessels within the corneal stroma. All acquisition settings were kept constant throughout the study to allow reliable comparison among experimental groups.

### 2.9. Network Pharmacology Analysis

The SMILES structure of Chi was first retrieved from PubChem. Potential targets of the compound were then predicted using multiple independent platforms, including PharmMapper [24], SEA [25], SuperPred [26], and TargetNet [27]. To obtain disease-related genes, the term “corneal neovascularization” was queried in OMIM [28], GeneCards [29], CTD [30], and DisGeNET [31]. Drug-associated and disease-associated targets were subsequently combined, duplicates were removed, and gene names were standardized to generate the final candidate target set.

Network topology evaluation was performed in Cytoscape 3.10.1 [32]. Targets with a degree value greater than the mean degree of the network were first selected. This set of degree-filtered targets was then used for Gene Ontology (GO) functional enrichment and Kyoto Encyclopedia of Genes and Genomes (KEGG) pathway analysis on the OmicShare Platform [33], using *Homo sapiens* as the reference species and Ensembl version 109 for annotation. In parallel, the maximal clique centrality (MCC) algorithm was applied to the same PPI network to further identify the most influential core targets [34].

### 2.10. Histological Staining

Whole eyes were carefully excised and immediately placed in 10% paraformaldehyde (PFA) for fixation at 4 °C overnight. After fixation, tissues were processed for either OCT cryo-embedding or paraffin embedding, depending on the subsequent histological requirements. Embedded samples were sectioned at a uniform thickness of 5 μm, and the prepared sections were stored at –80 °C until use.

Hematoxylin and eosin (H&E) staining was carried out with a commercial H&E staining kit according to the manufacturer’s protocol. Following completion of the staining procedure, corneal architecture and pathological alterations were examined using a bright-field optical microscope (Nikon, Tokyo, Japan), and representative images were recorded for comparative analysis among experimental groups.

### 2.11. Immunofluorescence Staining

Paraffin sections were first baked at 60 °C for 30 min, followed by deparaffinization and graded rehydration. Slides were immersed in xylene I and xylene II for 15 min each and then sequentially passed through 100%, 95%, 80%, and 70% ethanol for 5 min each. After rehydration, the sections were rinsed in PBS three times (5 min per wash). Antigen retrieval was performed using 1× antigen retrieval solution (Beyotime, Shanghai, China). Slides were heated in a microwave at high power for 10 min, cooled to room temperature, and washed again with PBS. Excess buffer around the tissue was removed using absorbent paper, and the tissue area was outlined with a hydrophobic barrier pen. Endogenous peroxidase activity was quenched by incubating the sections with 3% H_2_O_2_ for 20 min at room temperature, followed by PBS washing.

Tissues were then blocked with 2% BSA for 1 h at room temperature. After removing the blocking solution, slides were washed with PBS (3 × 5 min), and primary antibodies diluted according to the manufacturer’s instructions were applied. Sections were incubated overnight at 4 °C. The following day, the primary antibody solution was removed, and the slides were washed in PBS three times. Under light-protected conditions, fluorophore-conjugated secondary antibodies were added and incubated for 1 h at room temperature, followed by PBS washing. Nuclei were counterstained with DAPI (Sigma, St. Louis, MO, USA), and sections were mounted with antifade medium. The stained slides were covered with aluminum foil to protect them from light and stored at 4 °C until imaging. Fluorescence images were acquired using a Leica TCS SP8/880 laser confocal microscope (Leica Microsystems, Wetzlar, Germany).

### 2.12. Reverse Transcription and Quantitative PCR

Total RNA was isolated from tissue samples using TRIzol reagent (Takara Bio, Kusatsu, Japan) following the manufacturer’s protocol. Tissue samples were thoroughly homogenized with a mechanical tissue disruptor to ensure complete lysis. Reverse transcription was performed using the All-in-One RT SuperMix Perfect for qPCR (Vazyme, Nanjing, China) to generate cDNA from the extracted RNA. Quantitative PCR was conducted with ChamQ Universal SYBR qPCR Master Mix (Vazyme, Nanjing, China) on a real-time PCR system (Table 1). β-actin served as the endogenous control for normalization. Relative transcript levels were calculated using the 2−ΔΔCT method.

### 2.13. Western Blotting

Total protein was extracted from tissue samples using RIPA buffer supplemented with phosphatase and protease inhibitors. The proteins were separated on a 10% SDS-PAGE gel and transferred onto a nitrocellulose membrane. The membrane was blocked with 5% non-fat milk for 1 h and then incubated with primary antibodies at 4 °C overnight. After washing with TBST, the membrane was incubated with horseradish peroxidase-conjugated secondary antibodies at room temperature for 1 h. Protein bands were detected using the Immobilon Western HRP Substrate. Image acquisition was performed using ImageJ (v1.45g), and grayscale quantification was analyzed with GraphPad Prism (v9.5.0).

### 2.14. Safety Evaluation

Following anesthesia, the mouse eye was gently exposed and secured on the imaging platform. Optical coherence tomography (OCT) was used to record corneal and retinal structures. Fundus photography and fluorescein angiography were then performed with the OptoProbe system. Mydriasis was induced using a compound tropicamide–phenylephrine solution, and fundus images were captured with the optic disk positioned at the center. For angiography, 10% fluorescein sodium (0.0075 mL/g) was injected intraperitoneally, and imaging was carried out once dye circulation had stabilized. Identical magnification and exposure parameters were applied throughout all examinations to maintain consistency across time points.

### 2.15. Statistical Analysis

Data are presented as the mean ± standard error of the mean (SEM). Statistical analyses were performed using GraphPad Prism. Unpaired *t*-tests were applied for comparisons between two groups, whereas ANOVA was used for analyses involving more than two groups. A *p* value < 0.05 was considered statistically significant.

## 3. Results

### 3.1. Alterations in Oxidative Stress, VEGF Signaling, and PPAR Pathways After Alkali Burn and Chi Effects on Cell Viability

**Figure 1:**
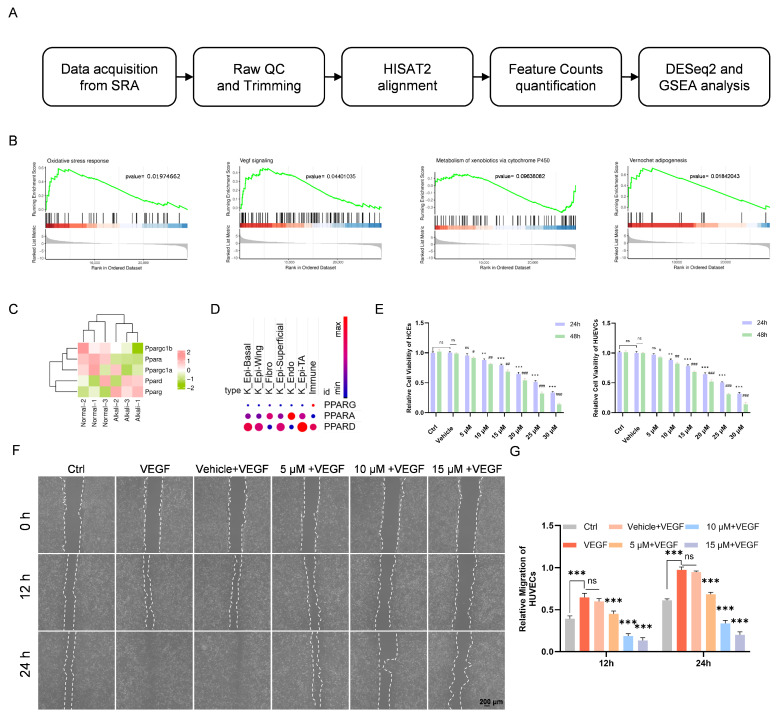
Effects of different dosages of Chi on cell migration and quantitative analyses. (**A**) Workflow of the data-mining strategy. The transcriptome data were downloaded from the SRA database. (**B**) Representative GSEA-enriched biological processes comparing alkali-burned corneas with healthy corneas. (**C**) Heatmap showing the expression profiles of PPAR isoforms and their coactivators in alkali-burned corneas. Colors represent normalized relative expression levels across samples. (**D**) Cellular distribution of the three PPAR isoforms in the normal cornea. The color scale shows the average expression of each gene in each cell cluster, with darker colors indicating higher expression. Immune: Immune cells; K_ Epi-TA: Corneal epithelium transit amplifying cells; K_ Endo: Corneal endothelium cells; K_ Epi-Superficial: Corneal superficial epithelium cells; K_ Fibro: Corneal fibroblasts; K_ Epi-Wing: Corneal wing epithelium cells; K_ Epi-Basal: Corneal basal epithelium cells. (**E**) Relative viability of HCEs and HUVECs treated with Chi for 24 or 48 h (*n* = 6 per group). (**F**) Images of scratch-wound assays in HUVECs following treatment with different dosages of Chi. (**G**) Quantitative analysis of scratch (*n* = 4 per group). ** *p* < 0.01, *** *p* < 0.001; ns, non-significant, for comparisons with the vehicle group; ^#^ *p* < 0.05, ^##^ *p* < 0.01, and ^###^ *p* < 0.001 for comparisons of relative cell viability between 24 and 48 h treatments.

To explore potential therapeutic targets following alkali burn, we performed GSEA on corneal transcriptome data obtained 7 days after injury (Figure 1A). The analysis showed that alkali burn markedly activated biological processes related to oxidative stress and VEGF signaling. Interestingly, alkali burn also induced remodeling of xenobiotic metabolism and energy metabolic networks mediated by the PPAR pathway (Figure 1B).

To further investigate the role of the PPAR family in alkali-burned corneas, we first compared the expression of PPAR-α, PPAR-δ, and PPAR-γ and their associated coactivators between alkali-burned and normal corneas using publicly available transcriptomic datasets. All showed varying degrees of expression changes, among which PPAR-γ displayed the most prominent alteration (Figure 1C).

We then examined the distribution of these three major isoforms across different cell populations using a single-cell transcriptomic dataset from the normal cornea (GEO: GSE199013) [21]. The results showed that PPAR-α and PPAR-δ were highly expressed in most corneal epithelial cells, whereas PPAR-γ exhibited low expression under homeostatic conditions (Figure 1D).

Given the potential involvement of the PPAR family in alkali burn, we next assessed the therapeutic profile of Chi, a PPAR agonist with predominant activity toward PPAR-γ [35]. Cell viability was evaluated in HCEs and HUVECs using CCK-8 following exposure to different concentrations of Chi. Both cell types exhibited dose- and time-dependent cytotoxicity, and 10 μM Chi was identified as a relatively safe concentration (Figure 1E). Based on prior studies, 20 ng/mL recombinant human VEGF was selected as the optimal concentration and was used for all subsequent cell experiments. In the scratch assay, Chi significantly reduced HUVEC migration at all tested concentrations compared with the VEGF-treated vehicle group (Figure 1F). Quantification showed that inhibition became evident at 5 μM Chi, reached its maximum at 10 μM Chi, and increased further but began to plateau at 15 μM Chi (Figure 1G).

### 3.2. Chi Exerts Anti-Angiogenic Activity in HUVECs and Reduces ROS in HCEs

**Figure 2:**
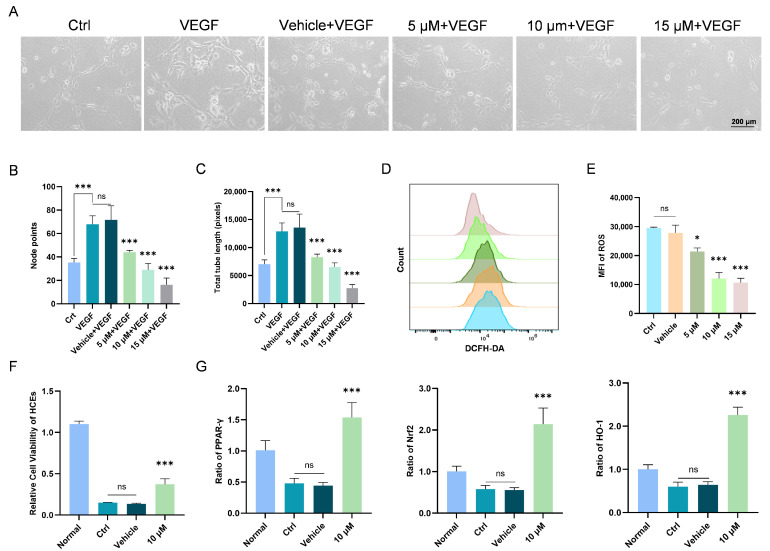
Effects of different concentrations of Chi on tube formation and intracellular ROS levels. (**A**) Tube formation patterns of HUVECs treated with different concentrations of Chi. (**B**) Quantification of node points in the tube formation assay (*n* = 4 per group). (**C**) Quantification of total tube length (*n* = 4 per group). (**D**) Flow-cytometric analysis of intracellular ROS levels after Chi treatment. Different colors represent different groups, and detailed group information is shown in (**E**). (**E**) Quantitative ROS fluorescence intensity (*n* = 3 per group). (**F**) Protective effect of 10 μM Chi on cell survival (*n* = 6 per group). (**G**) Detection of gene expression levels of anti-oxidative factors in vitro in each group. Nrf2, HO-1, PPAR-γ normalized to β-actin level (*n* = 6 per group). * *p* < 0.05, *** *p* < 0.001; ns, non-significant, comparison between the specified group and the vehicle group.

In the tube formation assay, Chi also suppressed the angiogenic capacity of HUVECs across all concentrations relative to the VEGF-treated vehicle group (Figure 2A). Analysis of average node number and total tube length demonstrated a concentration-dependent inhibitory effect, although the incremental reduction diminished at higher doses (Figure 2B,C).

ROS are highly reactive oxygen-containing molecules generated during cellular metabolism and are closely linked to oxidative stress responses [36]. Oxidative stress has been demonstrated to be an important mechanism inducing neovascular formation. Therefore, ROS also plays a key regulatory role in the initiation and progression of corneal neovascularization [37]. To evaluate the ROS-scavenging ability of Chi, we examined intracellular ROS levels under different concentrations of Chi by flow cytometry. The results showed that 5 μM, 10 μM, and 15 μM Chi all significantly reduced intracellular ROS levels, and considering cellular safety, 10 μM Chi appeared to be the most appropriate concentration (Figure 2D,E).

To assess whether Chi provides protection under oxidative stress, we measured cell viability using CCK-8 in HCEs treated with 10 μM Chi (Figure 2F). The results indicated that 10 μM Chi effectively increased cell survival under oxidative stress conditions.

To further evaluate the inhibitory effect of Chi on corneal oxidative stress, we used an H_2_O_2_-induced oxidative stress model in HCEs and detected the expression levels of Nrf2 and HO-1 by RT-PCR. The results showed that in the 10 μM Chi group, the transcriptional levels of PPAR-γ, Nrf2, and HO-1 were markedly upregulated (Figure 2G).

### 3.3. Chi Promotes Corneal Repair and Suppresses Neovascularization In Vivo

**Figure 3:**
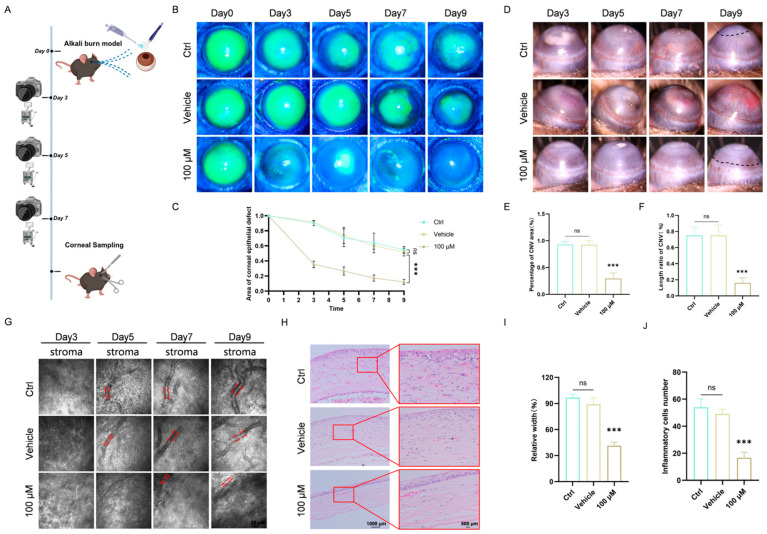
Chi effectively promotes corneal recovery and reduces neovascularization in vivo. (**A**) Experimental workflow. (**B**) Fluorescein sodium staining of the cornea. (**C**). Quantitative analysis of wound area (*n* = 6 per group). (**D**) Slit-lamp photographs showing corneal surface changes after treatment. (**E**) Statistical analysis of CNV area (*n* = 6 per group). (**F**) Statistical analysis of CNV length (*n* = 6 per group). (**G**) In vivo corneal confocal microscopy showing cellular and vascular morphology across different corneal layers. (**H**) H&E-stained corneal sections of treated mice. (**I**) Statistics of the relative width of blood vessels (*n* = 3 per group). (**J**) Quantitative analysis of inflammatory infiltration (*n* = 3 per group). *** *p* < 0.001, ns, non-significant, comparison between the specified group and the vehicle group.

To further assess the antioxidant and anti-angiogenic effects of Chi, a standardized corneal alkali burn model was established (Figure 3A). To comply with the ophthalmic formulation requirement of DMSO ≤ 0.1%, and considering dilution and penetration loss after topical instillation, 100 μM Chi was selected for in vivo application based on the effective in vitro concentration of 10 μM. The final dosing solution contained 0.1% DMSO.

Corneal epithelial repair was evaluated by fluorescein sodium staining, and neovascular growth was documented by slit-lamp imaging. The control and vehicle groups exhibited extensive epithelial defects and marked neovascularization, whereas 100 μM Chi promoted wound closure and markedly reduced new vessel formation (Figure 3B,D). Quantitative analysis of fluorescein staining further confirmed the significant enhancement of corneal healing in the 100 μM Chi group compared with the vehicle group (Figure 3C). Analysis of neovascular area and vessel length showed that 100 μM Chi significantly outperformed both parameters compared with the vehicle group (Figure 3E,F).

In vivo confocal microscopy provided additional visualization of the stroma. Compared with the vehicle group, 100 μM Chi significantly reduced the diameter of blood vessels in the corneal stroma (Figure 3G,I). H&E staining demonstrated that inflammatory infiltration was substantially alleviated in the 100 μM Chi group (Figure 3H,J).

Collectively, these findings indicate that Chi effectively promotes corneal wound repair and suppresses alkali burn-induced neovascularization in vivo.

### 3.4. Chi Demonstrates Comparable or Superior Efficacy to Selective PPAR-α or PPAR-γ Agonists

Fenofibrate was selected as a PPAR-α agonist at 200 μM in the mouse CNV model and has been reported to inhibit corneal neovascularization [15]. Pioglitazone was used as a PPAR-γ agonist and similarly reduced angiogenic responses and oxidative stress [38]. To ensure consistency and comparability, pioglitazone was therefore applied at a concentration of 200 μM.

**Figure 4:**
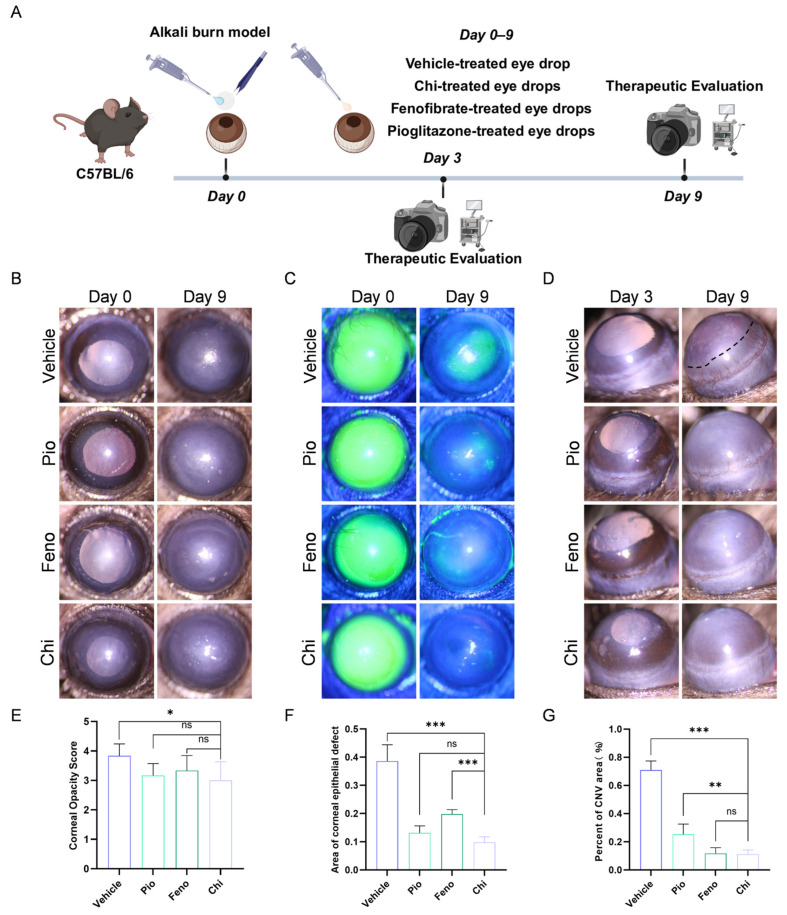
Comparison of the therapeutic effects of Chi, pioglitazone, and fenofibrate in a mouse corneal alkali burn model. (**A**) Therapeutic Evaluation Workflow. (**B**) Slit-lamp anterior segment images. (**C**) Representative fluorescein staining images. (**D**) Slit-lamp images showing corneal neovascularization. (**E**) Quantification of corneal opacity scores (*n* = 6 per group). (**F**) Quantification of corneal epithelial defect area after treatment (*n* = 6 per group). (**G**) Quantification of CNV area (*n* = 6 per group). * *p* < 0.05, ** *p* < 0.01, *** *p* < 0.001; ns, non-significant, comparison between the Chi group and the fenofibrate, pioglitazone, and vehicle group.

To assess the therapeutic performance of Chi eye drops in CNV, various eye drops were administered (Figure 4A). Slit-lamp examination showed that Chi improved corneal transparency compared with the vehicle group (Figure 4B). Quantitative analysis of corneal opacity scores showed no statistically significant differences among the Chi, fenofibrate, and pioglitazone groups (Figure 4E). Regarding the corneal injury area, Chi reduced the damaged area compared with the vehicle group (Figure 4C). No significant difference was detected between Chi and pioglitazone, whereas Chi showed better recovery than fenofibrate (Figure 4F). In terms of the corneal neovascularization area, Chi reduced the CNV area compared with the vehicle group (Figure 4D). Fenofibrate showed no significant difference relative to Chi, whereas Chi demonstrated stronger inhibitory effects than pioglitazone (Figure 4G).

Overall, the Chi group showed broader improvement across multiple corneal outcome measures than groups treated with PPAR-α or PPAR-γ agonists alone, underscoring the therapeutic advantage of multi-target drugs.

### 3.5. Network Analysis Reveals Key Genes and Signaling Pathways Targeted by Chi

**Figure 5:**
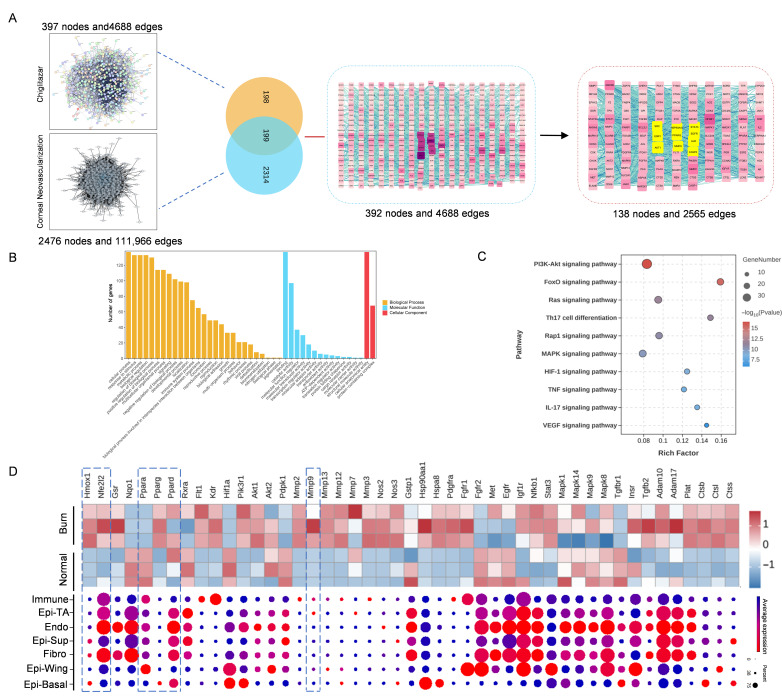
Integrative network analysis reveals potential targets of Chi. (**A**) Construction of the drug–disease–target network based on public databases. Different colors represent different levels of importance within the network, with highlighted targets indicating the core components. (**B**) GO enrichment analysis of the 99 targets with degree values above the mean. (**C**) KEGG pathway enrichment analysis of the 99 targets with degree values above the mean. (**D**) Expression and distribution of the 99 overlapping DEGs in the cornea. Upper: heatmap showing the 99 overlapped DEGs in alkali-burned corneas. Lower: dot plot showing the gene expression level and distribution in different corneal cells. The single-cell transcriptome data were downloaded from the GEO database: GSE199013. The color scale shows the average expression of each gene in each cell cluster, with darker colors indicating higher expression. Immune: Immune cells; Epi-TA: Corneal epithelium transit amplifying cells; Endo: Corneal endothelium cells; Epi-Sup: Corneal superficial epithelium cells; Fibro: Corneal fibroblasts; Epi-Wing: Corneal wing epithelium cells; Epi-Basal: Corneal basal epithelium cells.

We applied a network pharmacology approach to map Chi’s regulatory network. Integration of predicted drug targets with CNV-related genes identified 199 overlapping genes, from which 99 nodes were selected using the mean degree threshold, and 10 core targets were determined by the MCC algorithm. Analysis of the MCC-ranked core targets revealed that PPAR-γ occupied the most central position within the network, whereas PPAR-α showed moderate connectivity and PPAR-δ exhibited only limited network centrality (Figure 5A).

GO enrichment of the 99 genes demonstrated that the top 10 biological processes were enriched in cellular processes, responses to stimuli, metabolic processes, biological regulation, positive and negative regulation of biological processes, multicellular organismal processes, signaling, interspecies interaction-related biological processes, immune system processes, developmental processes, localization, and locomotion (Figure 5B). KEGG pathway enrichment indicated that the predominant pathways involved PI3K–Akt, FoxO, Ras, Th17 cell differentiation, Rap1, MAPK, HIF-1, TNF, IL-17, and VEGF signaling (Figure 5C).

To further refine targets prioritized by network pharmacology, we integrated published transcriptomic data from alkali-burned corneas with a single-cell atlas of the normal cornea to examine the expression patterns of the 99 candidate targets. Nrf2, HO-1, and MMP-9 showed high expression levels in alkali-burned corneas compared with healthy controls (Figure 5D). Given its prominent expression in corneal epithelial cells (Figure 5D) and close relevance to the cornea, HO-1 was prioritized for further validation. Nrf2 was included because it is the canonical upstream regulator of HO-1.

### 3.6. Chi Activates PPAR-α and PPAR-γ to Regulate Downstream Oxidative and Angiogenic Pathways

**Figure 6:**
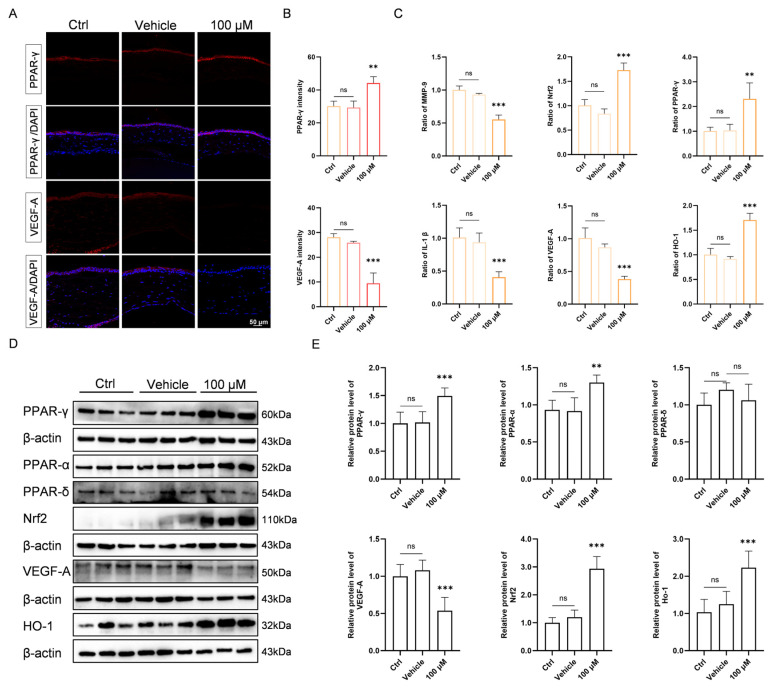
Validation of the predicted targets. (**A**) Immunofluorescence staining of VEGF-A and PPAR-γ in corneal tissue. (**B**) Quantitative analysis of fluorescence intensity (*n* = 3 per group). (**C**) Gene expression levels of angiogenic and oxidative stress-related factors in corneal tissue, including VEGF-A, PPAR-γ, MMP-9, IL-1β, Nrf2, and HO-1, were normalized to β-actin (*n* = 6 per group). (**D**) Western blotting analysis of PPAR-α, PPAR-γ, PPAR-δ, Nrf2, VEGF-A, and HO-1 in corneal protein extracts. (**E**) Quantification of protein band intensities (*n* = 6 per group). ** *p* < 0.01, *** *p* < 0.001; ns, non-significant, comparison between the specified group and the vehicle group.

To validate the predicted key targets, immunofluorescence was performed to examine the protein expression and spatial distribution of PPAR-γ and VEGF-A in the cornea. The results showed that corneal PPAR-γ expression was markedly increased in the 100 μM Chi group, whereas VEGF-A signals were markedly reduced (Figure 6A). To further assess the anti-angiogenic effect of Chi, lectin immunofluorescence staining was performed on corneal tissues. Compared with the vehicle group, lectin fluorescence intensity was markedly reduced after Chi treatment (Figure S1A,B).

Notably, the change in PPAR-γ was mainly confined to the corneal epithelium. Quantitative analysis confirmed the significance and consistency of these differences (Figure 6B). We next assessed the expression of Nrf2, HO-1, and related inflammatory and angiogenic factors by RT-PCR. Treatment with 100 μM Chi upregulated Nrf2/HO-1 and suppressed VEGF-A, IL-1β, and MMP-9 expression (Figure 6C).

Western blotting was used to examine the three PPAR isoforms as well as the downstream factors Nrf2, HO-1, and VEGF-A. Chi activated PPAR-α and PPAR-γ, while PPAR-δ activation appeared less prominent under the present model, dose, and exposure conditions. Antioxidant markers were increased, and VEGF-A was reduced (Figure 6D). Quantitative analysis supported the consistency and reliability of these findings (Figure 6E).

### 3.7. Topical Application of Chi Demonstrated Good Ocular Safety

**Figure 7:**
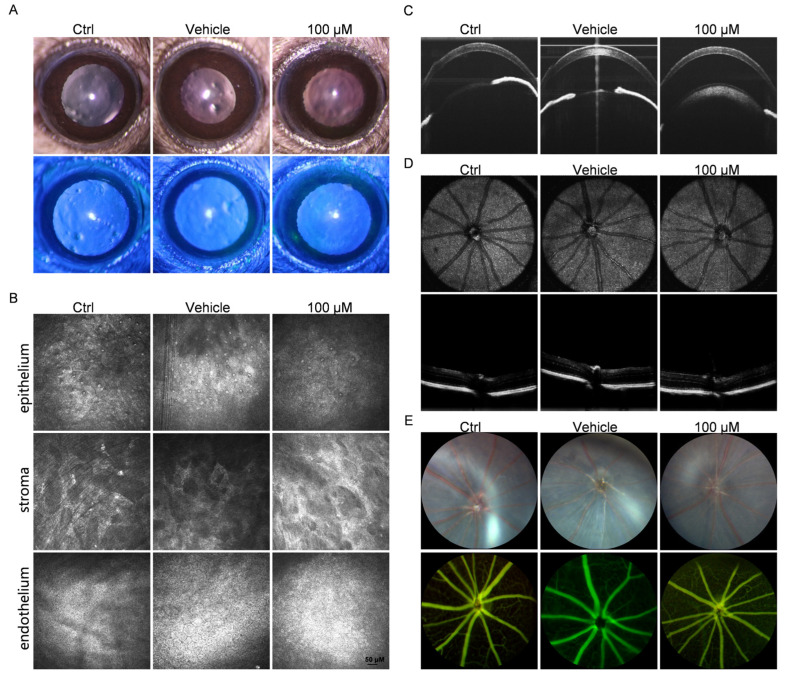
Evaluation of ocular safety. (**A**) Slit-lamp examination. (**B**) In vivo confocal microscopy showing the cellular morphology of the corneal epithelium, stroma, and endothelium. (**C**) Anterior segment OCT images showing corneal structure and morphology. (**D**) Retinal OCT imaging. (**E**) Fundus fluorescein angiography.

The therapeutic effect of Chi on CNV has been explored, but its safety under topical administration still requires further confirmation. Therefore, OCT imaging, fundus fluorescein angiography, and in vivo corneal confocal microscopy were performed nine days after treatment to systematically evaluate its impact on ocular tissues. Fluorescein staining results showed no abnormal staining after topical application of 100 μM Chi, indicating that the corneal surface was not irritated or damaged by the drug (Figure 7A).

OCT results showed that corneal layer organization remained normal in the Chi-treated group, and no significant differences in corneal thickness were observed among the groups (Figure 7C and Figure S2A). Fundus photography and fluorescein angiography were used to examine retinal morphology and vascular permeability. No leakage, neovascularization, or abnormal vascular patterns were detected in any group, based on either fundus images or fluorescein angiography (Figure 7D,E). No significant differences in retinal thickness were observed among the groups (Figure S2B). In addition, in vivo corneal confocal microscopy results showed that corneal cells in the 100 μM Chi group displayed regular morphology, clear borders, and cell density and arrangement comparable to those of normal corneas, with no evidence of damage or inflammation (Figure 7B).

## 4. Discussion

The cornea plays a critical role in maintaining visual clarity and shielding the eye from infection and injury [39]. Once CNV develops, this transparent refractive surface becomes compromised, and the cornea’s immune-privileged state is disrupted [40]. Although corticosteroids [41], non-steroidal anti-inflammatory drugs [41], and anti-VEGF agents [42] remain the standard pharmacologic options, each carries inherent limitations that constrain sustained use and long-term disease control [43]. Compared with conventional anti-VEGF therapies that mainly target VEGF-related signaling pathways, PPAR-targeted approaches can also regulate oxidative stress and inflammatory responses and have therefore gained increasing attention in CNV research [44]. Its broader mode of action suggests that it may be useful for patients who respond poorly to anti-VEGF therapy, and it may also work well in combination with anti-VEGF agents to reduce injection frequency, treatment burden, and cost. Nonetheless, targeting an individual PPAR isoform may not adequately address the complex and multifactorial processes driving CNV, underscoring the need for agents capable of engaging multiple PPAR subtypes simultaneously. Chi, a next-generation pan-PPAR agonist with multi-subtype activation properties [45], represents a promising candidate for such an approach. In this study, we demonstrated that Chi exerted robust anti-neovascular and tissue-protective effects in both cellular and animal models of CNV. In addition, we conducted preliminary analyses to characterize its potential activation pattern, thereby laying the groundwork for more detailed mechanistic investigations.

In CNV, all three PPAR isoforms are recognized for their roles in mitigating oxidative stress and inflammation [16,46]. Notably, agonists of PPAR-γ and PPAR-α have shown clear anti-neovascular effects in multiple in vitro and in vivo studies [46,47], whereas activation of PPAR-δ has been reported in some contexts to exert an opposite influence [16,48]. Therefore, as a new pan-PPAR agonist, the therapeutic significance of Chi in CNV remains uncertain and requires further verification. In vitro, Chi exerted significant anti-angiogenic and anti-oxidative effects at 10 μM. However, topical ocular delivery is subject to rapid precorneal clearance and limited corneal penetration, which can markedly reduce drug exposure in corneal tissues. Therefore, a higher concentration (100 μM) was used in vivo to ensure adequate local exposure. In the mouse CNV model, this dose produced beneficial therapeutic effects and was well tolerated, suggesting the potential of Chi for further translational development. Further studies on ocular pharmacokinetics and tissue distribution are still needed to support its clinical application. Notably, compared with fenofibrate and pioglitazone, Chi showed differential advantages across outcome measures: it performed better than fenofibrate in corneal repair-related parameters and had stronger effects than pioglitazone in vascular inhibition-related outcomes. However, these comparisons are based on the current dosing and administration conditions. Given possible differences in dose selection, pharmacological properties, and ocular bioavailability among the three agents, further studies will be required to assess the relative advantages and translational potential of Chi.

We further established a structured “disease–gene–target–drug” analytical framework to explore its mechanism in a cost-efficient and streamlined manner [49]. The analysis suggested that the major regulatory effects of Chi in CNV are likely mediated through PPAR-α and PPAR-γ, whereas PPAR-δ ranked lower in predicted activation. Subsequent experimental results provided preliminary support for this observation. Regarding the insufficient activation of PPAR-δ, we consider that several factors may contribute to this observation. First, although the concentration of the eye drops is relatively high, the residence time on the ocular surface is limited. Factors such as mouse blinking [50] and the corneal barrier [51] may reduce the effective local exposure, making it insufficient to fully activate PPAR-δ. Second, previous studies have shown that Chi exhibits the weakest activation toward PPAR-δ in different cell lines, including SMMC-7721 and U2OS cells [52]. Based on this characteristic, we speculate that the low activation of PPAR-δ in mouse corneal tissue may similarly reflect its inherently limited responsiveness to Chi. In the pathway prediction analysis, the results appeared overly broad because of the complexity of the involved signaling networks. We therefore attempted to identify common elements across the diverse pathways. By integrating prior studies [53,54,55] with our experimental findings, we observed that multi-target activation ultimately converged on two critical processes: the regulation of oxidative stress and VEGF-A angiogenic signaling. PPAR-γ has been shown to directly regulate the expression of VEGF-A [56], and VEGF-A itself is markedly influenced by ROS levels [57]. Thus, the multi-target activation profile of Chi in CNV may exert its effects by jointly modulating these two key pathways. In view of prior reports indicating a possible link between PPAR-γ agonists and macular edema [58], despite the inconsistency of existing evidence [59], we incorporated a comprehensive assessment of fundus and ocular surface safety into the present study. No discernible retinal structural alterations and ocular surface adverse responses were observed in the Chi-treated group, supporting the ocular tolerability of Chi and underscoring its suitability for further translational investigation.

Despite these encouraging findings, several limitations should be acknowledged. First, this study relied on an animal model of corneal neovascularization, and its translational relevance to human CNV requires further validation. Second, although Chi exhibited notable therapeutic efficacy, it was not directly compared with current standard treatments for CNV, leaving its relative advantages within the existing therapeutic landscape unresolved. In addition, the long-term safety profile and durability of its therapeutic effects remain to be determined. Moreover, Chi has poor aqueous solubility, and DMSO was required as a solvent in the present experiments. Although the percent of DMSO was strictly controlled, the possibility of solvent-related interference cannot be fully excluded. This highlights the need to develop delivery systems or formulations better suited for ophthalmic administration. Finally, our conclusions on PPAR isoform contributions are primarily associative, based on expression and network analyses. Future isoform-specific inhibition or knockdown studies are needed to confirm causality.

This study identifies Chi as a promising therapeutic candidate for CNV, with potential significance for improving the quality of life of patients affected by this severe condition. Further investigations will be needed to translate these findings into clinical practice and to bring new and effective interventions to CNV management.

## 5. Conclusions

This study demonstrates that Chi alleviates oxidative stress and VEGF-A production in CNV by activating the PPAR-α and PPAR-γ. These results support further investigation of Chi for clinical translation in the management of CNV.

## Figures and Tables

**Table 1:** Primers used for RT-PCR.

Gene	Forward Sequence (5′−3′)	Reverse Sequence (5′−3′)
*m-β-actin*	CCTAAGGCCAACCGTGAAAAG	AGGCATACAGGGACAGCACAG
*h-β-actin*	ACAGAGCCTCGCCTTTGC	GCGGCGATATCATCATCC
*m-HO-1*	ACCTGACACAGTTCCCTTAC	GATTTGGGCTGCTGGTTCCA
*m-Nrf2*	TCACACCAGCTCTTTGGAGT	CTCGGGTGTCCTCTAAGCAA
*h-HO-1*	AAATTTCAGAAGGGCCAGGT	GACGACTGGGCTCTCCTTGT
*h-Nrf2*	TCTGACTCCGGCATTTCACC	AGGCCAAGTAGTGTGTCTCT
*m-PPAR-γ*	CTCGGAGGGCCAAGGATTCA	GGCAGTCTCCACTGAGAATA
*h-PPAR-γ*	ATAGATCCAGTGGTTGCAGA	ACTGCCATGAGGGAGTTGGA
*m-MMP-9*	CCACCGAGCTATCCACTCAG	CCCTAACGCCCAGTAGAGAT
*m-IL-1β*	GAAGAAGAGCCCATCCTCTG	GAAGAAGCCCCCCCTCTG
*m-VEGF-A*	CCCTTCGTCCTCTCCTTAAC	AGGAAGGGTAAGCCACTCCC

## Data Availability

The original contributions presented in this study are included in the article. Further inquiries can be directed to the corresponding authors. The public datasets used for the bioinformatics analysis are available at the Gene Expression Omnibus (GEO) database (https://www.ncbi.nlm.nih.gov/geo/, accessed on 1 January 2024).

## Supplementary Materials

The following supporting information can be downloaded at: https://www.mdpi.com/article/10.3390/antiox15040449/s1. Figure S1: (A) Immunofluorescence staining of Lectin in corneal tissue. (B). Quantitative analysis of fluorescence intensity (*n* = 3 per group). *** *p* < 0.001; ns, non-significant, comparison between the specified group and the vehicle group. Figure S2: (A). Quantitative analysis of corneal thickness (*n* = 3 per group). (B) Quantitative analysis of retinal thickness (*n* = 3 per group). ns, non-significant, comparison between the specified group and the vehicle group.
