# Supplementary material for: Chiglitazar Activates PPAR-α/γ to Suppress Oxidative Stress and Angiogenesis in Corneal Neovascularization
Source: Antioxidants (Basel). 2026 Apr 2;15(4):449. doi: 10.3390/antiox15040449 (PMC13113329; doi:10.3390/antiox15040449)
Supplement: Supplementary file 1 [file antioxidants-15-00449-s001.zip › Figure S1.pdf]

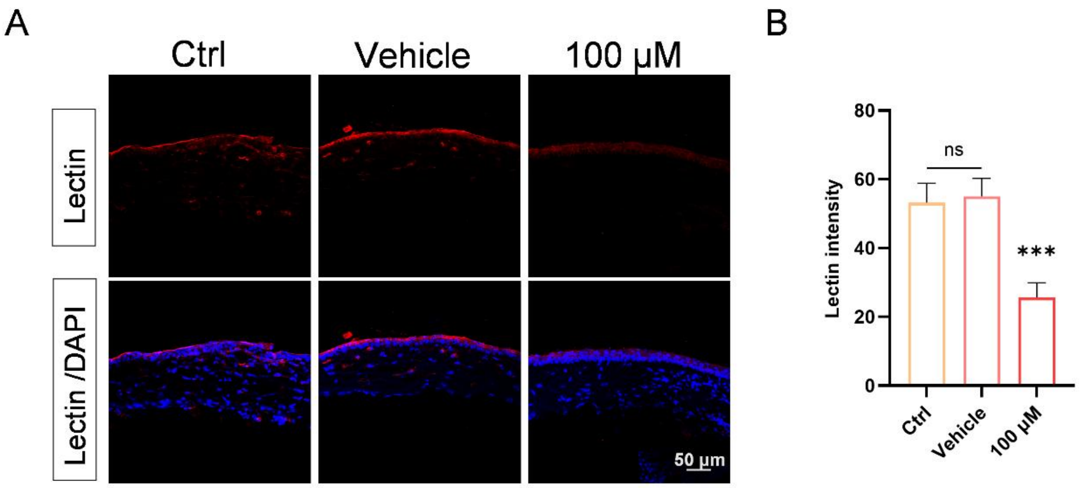

Figure S1. (**A**) Immunofluorescence staining of Lectin in corneal tissue. (**B**). Quantitative analysis of fluorescence intensity ($n = 3$ per group). *** $p < 0.001$; ns, non-significant, comparison between the specified group and the vehicle group
