# Supplementary material for: Chiglitazar Activates PPAR-α/γ to Suppress Oxidative Stress and Angiogenesis in Corneal Neovascularization
Source: Antioxidants (Basel). 2026 Apr 2;15(4):449. doi: 10.3390/antiox15040449 (PMC13113329; doi:10.3390/antiox15040449)
Supplement: Supplementary file 1 [file antioxidants-15-00449-s001.zip › Figure S2.pdf]

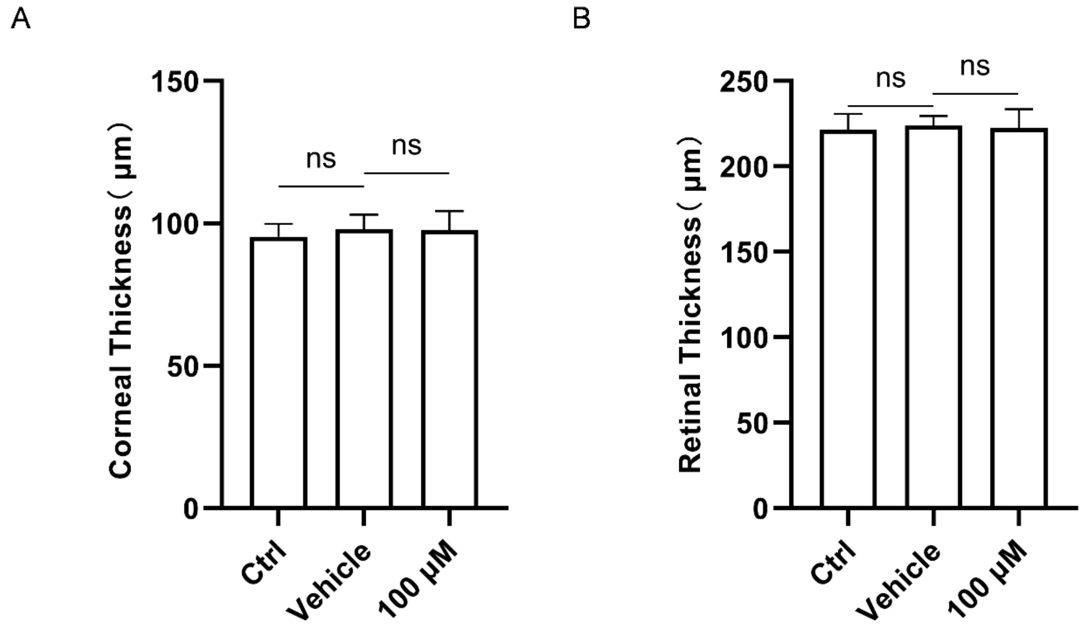

Figure S2. (**A**). Quantitative analysis of corneal thickness (*n* = 3 per group). (**B**) Quantitative analysis of retinal thickness (*n* = 3 per group). ns, non-significant, comparison between the specified group and the vehicle group.
